# The Zygomatic Anatomy-Guided Approach, Zygomatic Orbital Floor Classification, and ORIS Criteria—A 10-Year Follow-Up

**DOI:** 10.3390/jcm12206681

**Published:** 2023-10-23

**Authors:** Rafal Zielinski, Jakub Okulski, Wojciech Simka, Marcin Kozakiewicz

**Affiliations:** 1 Stomatologia na Ksiezym Mlynie, 16D Tymienieckiego, 90-365 Lodz, Poland; 2 Department of Maxillofacial Surgery, Medical University of Lodz, 113st Zeromskiego, 90-001 Lodz, Poland; jakub.okulski@gmail.com (J.O.); marcin.kozakiewicz@umed.lodz.pl (M.K.); 3 Faculty of Chemistry, Silesian University of Technology, 44-100 Gliwice, Poland; wojciech.simka@polsl.pl

**Keywords:** zygomatic implants, ZOF classification, ORIS criteria, ZAGA classification

## Abstract

Background: Presently, the management of patients with maxilla bone defects of the Cawood V or VI class is achieved using zygomatic or individual implants or through augmentation of the bone. For zygomatic implants, the ORIS criteria represent the most common factor in helping practitioners register success rates. The zygomatic anatomy-guided approach (ZAGA)and zygomatic orbital floor (ZOF) are factors that are crucial to examining the anatomy of a particular patient before the procedure. The aim of thisarticle is to find the statistical relationship between the abovementioned terms and other factors. Methods: A total of 81 patients underwent zygomatic implant procedures in different configurations. The ORIS, ZAGA, and ZOF parameters were compared with other factors such as type of surgery, sex, age, and the anatomy of the zygomatic bone. Results: Most patients in this article were classified as ZAGA Class 2. The relationships between type of surgery and ZAGA classification, and ZAGA and sinus/maxilla zygomatic implant localization were statistically significant. Conclusions: The ZAGA and ZOF scales are practical and valuable factors that should be taken into account before surgery, whereas to date, criteria better than the ORIS scale have not been described in terms of the success of zygomatic implants. The ZOF scale might omit perforation of the orbit because this parameter warns a practitioner to be aware of the anatomy of the orbit.

## 1. Introduction

For many years, zygomatic implants have been utilized for the restoration of atrophic maxillae and the repair of inborn and acquired deficiencies in the maxillary region [1]. Presently, the management of patients with maxilla bone defects of the Cawood V or VI class is achieved using zygomatic or individual implants or via augmentation of the bone [2,3,4,5]. The main advantage of zygomatic implants is that there is no need to prepare much for the surgery since they are in stock, contrary to individual implants. However, surgery for the treatment of edentulism by means of individual implants can be performed under local anesthesia, whereas zygomatic implants are much safer when performed under general anesthesia. In practice, this makes zygomatic implants less available. Recently, high technology has enabled the manufacture of individual implants not only in titanium but also for screw-retained bridges, with the possibility ofimmediate loading. The augmentation of bone often demands a long time for the healing of the bone with many restrictions. A comparison of zygomatic implant treatment with conventional implants in augmented bone clearly showed that the former led to fewer prosthodontic problems, better implant survival rates, a faster possibility of having a permanent bridge, and higher patient acceptance [6]. For zygomatic implants, the ORIS criteria represent the most common factor in helping practitioners register success rates [7]. The zygomatic anatomy-guided approach (ZAGA) and zygomatic orbital floor (ZOF) arefactors that are crucial to examining the anatomy of a particular patient before the procedure [8]. The aim of this article is to find the statistical relationship between the abovementioned terms and other factors such as type of surgery, height of the zygomatic bone, gender, and age.

## 2. Materials and Methods

### 2.1. Patients

Eighty-one patients with an average age of 53 years (ranging from 54 to 70 years, standard deviation = 8.65) and suffering from moderate to severe maxilla atrophy underwent surgical procedures between 2010 and 2017 at a privately owned dental implant clinic named Stomatologia na Ksiezym Mlynie in Lodz, Poland. All the patients were non-smokers, generally healthy, and had neither diabetes mellitus nor any other comorbidities, and this had a positive influence on the success rate of their implants.

Follow-up check-up visits were conducted until 2023. For each patient, cone beam computed tomography (CBCT) scans were taken both before and after the surgery. Notably, no templates or planning software were utilized during the procedures. All implants were inserted manually without the aid of guides.

The surgeries were performed by a single operator who lacked specialization in maxillofacial surgery but possessed significant expertise in dental implants, including extensive training in zygomatic implants from various international courses. Temporary screw-retained acrylic bridges were provided within 24 h for all patients. After 5 months, permanent bridges—acryl fused to titanium, porcelain fused to CoCr, or ZrOfully contoured on titanium suprastructures—were prepared. All suprastructures were milled using an HSC 20 Ultrasonic 5-axis machine (DMG MORI, Antoniusstr. 14; DE-73249 Wernau) at the wellCAMdent Milling Center (93-231 Dostawcza 14, Lodz, POLAND).

**Figure 1:**
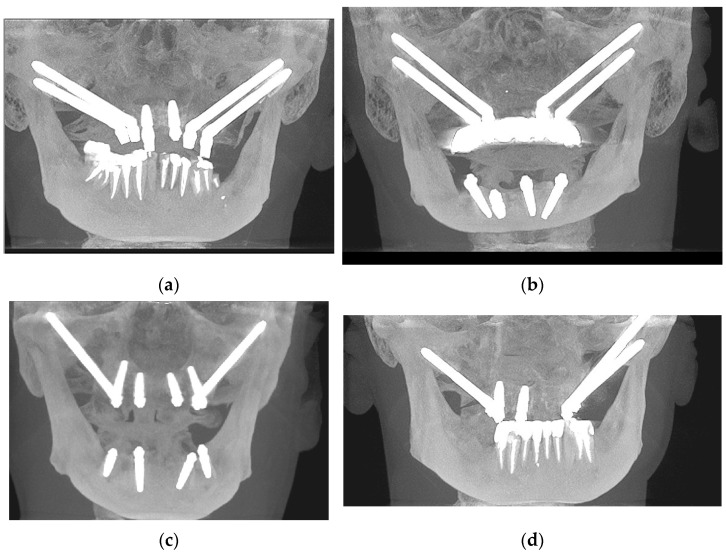
Some representative CBCT photos for each group: (**a**) Group 1—4 zygomatic implants + 1 or 2 conventional implants; (**b**) Group 2—4 zygomatic implants; (**c**) Group 3—2 zygomatic + 4 conventional implants; (**d**) Group 4—3 zygomatic + 1 or 2 conventional implants.

The patient cohort was categorized into four groups based on the distinct configurations of zygomatic implants:

1. Group 1 (14 patients) received 4 zygomatic implants along with 1 or 2 conventional implants.
2. Group 2 (15 patients) received 4 zygomatic implants exclusively.
3. Group 3 (46 patients) received 2 zygomatic implants and 4 conventional implants.
4. Group 4 (6 patients) received 3 zygomatic implants along with 1 or 2 conventional implants (as shown in Table 1). Figure 1.

### 2.2. Surgery

All patients took 1 g of Penicillin 24 h before surgery and then 1 pill every 12 h for 7 days. Surgeries were performed under general anesthesia with nasal intubation, supplemented by local anesthesia using articaine with epinephrine (Citocartin 200 14 mL). All patients were discharged from the dental clinic on the same day of the surgery. The preferred technique was the “sinus slot approach,” as described by Stella and Warner in 2000, which focuses on minimizing implant penetration into the maxillary sinus [4].

The surgical process commenced with a crestal incision spanning from one maxillary tuberosity to the opposite side. By creating a palatal flap, both the alveolar process and hard palate were exposed. Continuing the dissection along the infra-zygomatic crest toward the zygomatic bone (ZB), the surgeon gained access to the zygomatic region using a reverse Langebeck hook while carefully identifying the infraorbital nerve. Subsequently, a precise 4 cm × 3 cm window was carefully formed in the upper lateral aspect of the sinus wall, aligned with the extension of the infra-zygomatic crest, utilizing a diamond round bur. Following the reflection of the sinus mucosa, direct visibility of the sinus roof was achieved, facilitating the precise identification of the optimal point for drilling into the zygomatic bone. The drilling for the zygomatic implants was performed using an implant handpiece at a speed of 600 rpm, penetrating the crestal bone at a predetermined entry point. The operator could visualize the procedure through CBCT focused on the zygomatic and orbital regions.

All zygomatic implants used were Nobel Biocare 45° zygomatic implants with TiUnite surface (Nobel Biocare AB, Gothenburg, Sweden), with a diameter of 4.3. The lengths of all implants were documented. The duration of the surgery was measured from the time the patient fell asleep to the moment of awakening.

The spatial relationship between the location of the zygomatic implant and both the maxilla and the sinus was carefully examined for all patients. The number of zygomatic implants was recorded for different regions (16, 13, 23, 26), taking into account whether they were positioned intra- or extrasinusally, as well as intramaxillarily or extramaxillarily. Throughout the surgical procedures, the operator stood on the right side of the patient’s head, assisted by either a dental assistant or another doctor.

The ORIS scale was individually measured for each zygomatic implant. Simple regression testing was conducted to establish the correlation between the height and width of the zygomatic bone. Additionally, the height and distance between two zygomatic implants within the zygomatic bone were analyzed using the Mann–WhitneyU test.

### 2.3. CBCT Standardization

A CBCT scanner with a flat-panel detector was used in all cases (i-Cat; Imaging Sciences International, LLC, Hatfield, PA, USA). The voxel size was 0.2 mm × 0.2 mm × 0.2 mm. The exposure volume was set at 0.4 mm. Manufacturer-recommended settings of 80 kV and 5 mA were employed. The Frankfurt plane was used rather than the occlusal plane. The scan range was from the supraorbital edge to the mandible. In any patient, preoperative planning with Nobel Clinician software ver. 1.5 was used. 

### 2.4. Statistical Analysis

Statgraphics Centurion version 18.1.12 (StatPoint Technologies, Warrenton, VA, USA) was used for statistical analyses. Statistical tests were Mann–Whitney U, Kruskal–Wallis, ANOVA, and R-squared. The detected relationships were assumed to be statistically significant when *p* < 0.05.

## 3. Results

### 3.1. ZAGA

**Figure 2:**
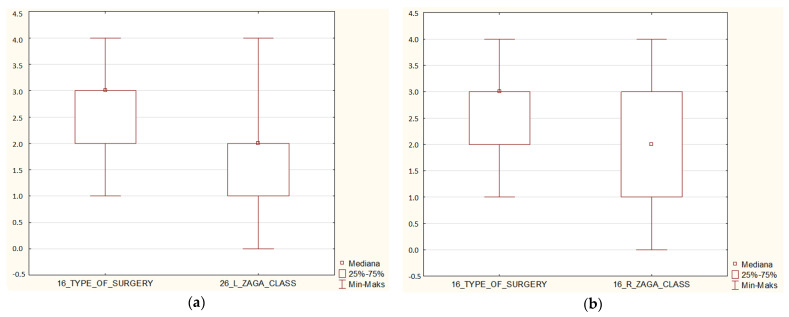
Depending on the type of surgery − Group 1: 4 zygomatic implants + 1 or 2 conventional implants; Group 2: 4 zygomatic implants; Group 3: 2 zygomatic + 4 conventional implants; Group 4: 3 zygomatic + 1 or 2 conventional implants ZAGA class was registered. In groups I and II, where the atrophy of the maxilla was the highest, the ZAGA classification was the lowest. The above relationship was statistically significant (*p* < 0.05). On (**a**) the right side and (**b**) the left side of the maxilla, the relationship between the type of surgery and the ZAGA class was statistically significant.

**Figure 3:**
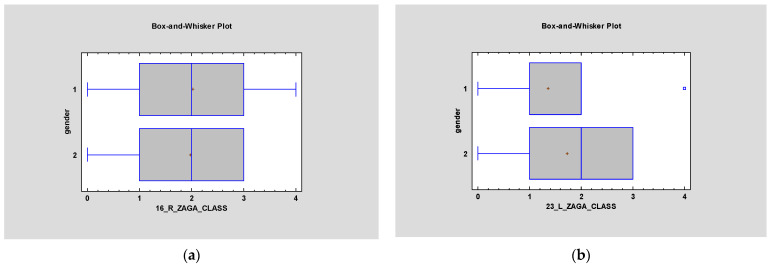
Despite the fact that there is a difference in face morphology in men and women, there is no statistical significance in ZAGA class and gender (*p* > 0.5) on the right side (**a**) and left side (**b**).

**Figure 4:**
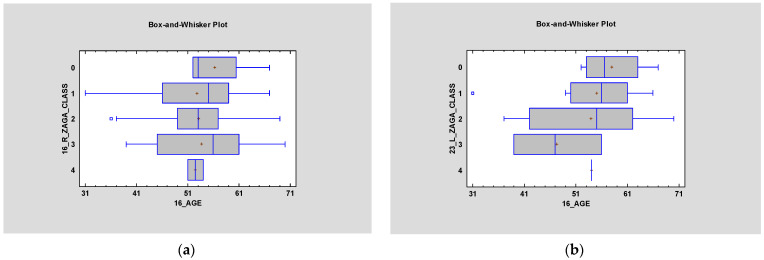
Box and whisker plots showing the relationship between age and ZAGA class on the right side (**a**) and left side (**b**). Neither show statistical significance (*p* > 0.5).

Most patients described in the article had ZAGA Class 2 on both sides—right average 2.0, and left average 1.77. The relationship between type of surgery and ZAGA classification was found to bestatistically significant on both sides (*p* < 0.05) (Figure 2). However, *p* > 0.05 in terms of ZAGA and gender (Figure 3). Simple regression showed an interesting relationship between ZAGA, sinus, and maxilla because the left sidewas statistically significant (*p* < 0.05). The relationship between ZAGA and intra-/extramaxillary was statistically significant, whereas no statistical significance was observed in the relationship between ZAGA and intra-/extrasinus. On the right side, however, it was the opposite: there was a statistically significant relationship between ZAGA and intra-/extrasinus, and no statistical significance (*p* > 0.05) was observed between ZAGA and intra-/extramaxillary. The box and whisker plots showed a relationship between age and ZAGA class but without statistical significance(*p*> 0.5) (Figure 4).

### 3.2. ORIS

**Figure 5:**
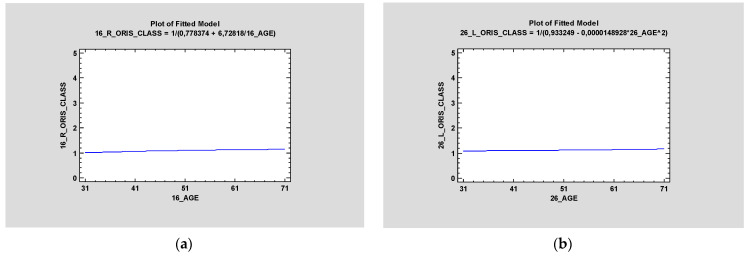
Simple regression proved no statistical significance between ORIS on zygomatic implants in Region 16 (**a**) and Region 26 (**b**) and age (*p* > 0.5).

ORIS criteria were registered for all four zygomatic implants, and the average values for regions 16, 26, 13, and 23 were 1.28,1.31,1.33, and1.45, respectively. The ANOVA test between ORIS and age proved there was no statistically significant relationship (*p* > 0.5) between those values in any region of the zygomatic implant—16, 13, 23, or 26 (Figure 5).

**Figure 6:**
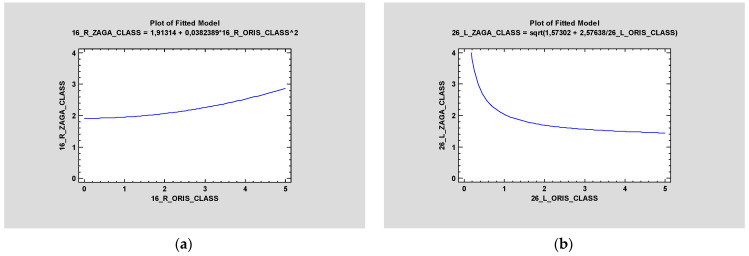
Simple regression proved no statistical significance between ORIS and ZAGA class on the right side (**a**), and the squared model with the same parameters on the left side (**b**) also proved no statistical significance (*p* > 0.05).

In the article, the relationship between ZAGA class and ORIS for each zygomatic implant was investigated, and the ANOVA test proved there was no statistical significance between these criteria. Regardless of the ZAGA class, the ORIS scale was an independent factor(Figure 6).

A Mann–Whitney U test showed no statistical relationship between the ORIS scale and both intra or extrasinus or maxillary implant placement (*p* > 0.05). 

### 3.3. ZOF

**Figure 7:**
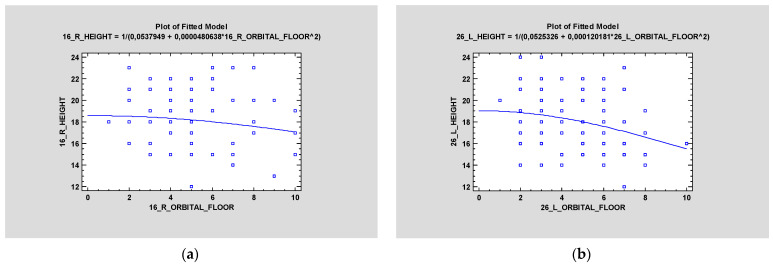
Simple regression was made to check the height of the zygomatic bone on the left side and the zygomatic orbital floor (ZOF) (in mm). It was statistically significant (*p* < 0.05) (**b**). The same test proved a lack of statistical significance (*p* > 0.05) on the right side (**a**).

**Figure 8:**
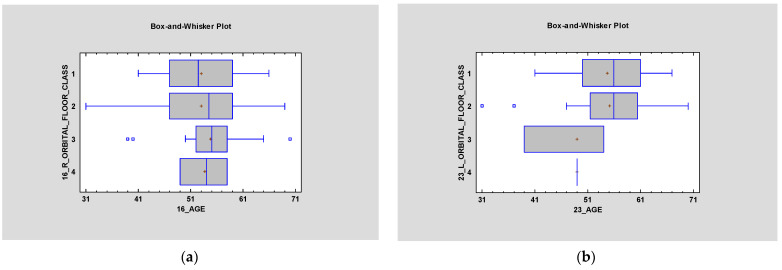
Box and whisker plots were used to check if ZOF classification depends on age (*p* > 0.05) on both sides (left—(**a**) and right—(**b**)). It was proved to have no statistical significance (*p* > 0.05).

Distance between zygomatic implants in the zygomatic bone and ZOF (zygomatic orbital floor). Classification is also statistically irrelevant on the right side; however, *p* < 0.05 on the left side (Figure 7). It was proved that ZOF classification does not depend on age—statistical significance was higher than 0.05 (*p* > 0.05) on both sides (Figure 8).

## 4. Discussion

### 4.1. Success Criteria of Conventional Implants

The criteria of success for all dental implants were first suggested by Schnitman and Shulman in 1979. They determined that the following factors are crucial in assessing thesuccess rate of dental implants: stability, peri-implant radiolucency, crestal bone level with respect to platform, peri-implant gingival health, and long-term survival rate. They used mobility as a criterion for success, which later was found to be a mistake [9]. 

Then, in 1986, Albrektsson et al. suggested criteria of success that have been considered to bethe reference for 30 years [10]. They clearly stated that success depends on the lack of peri-implant radiolucency. They specified as tolerable a loss of vertical bone height maximally of 0.2 mm per year. In that article, the authors described bone loss after implant in only the first year. 

Subsequently, in 1994, Albrektsson and Isidor stated that one of the requirements for considering the implant procedure successful was maintaining a marginal crestal bone loss of under 1 mm within the initial year of implant usage [11]. These prerequisites must be met without any iatrogenic involvement, and the implant’s long-term survival rate should be at least 85% after 5 years and 80% after 10 years.

The consensus document from the Toronto Osseointegration Conference in Clinical Dentistry retained the criteria put forth by Albrektsson and Isidor in 1994 [10,11,12]. However, an additional subjective factor was introduced: the requirement for a functional and visual factor of dental prosthesis that satisfies both the patient and the dentist. There was also a discussion about using a standardized periapical radiographic technique to assess and compare levels of crestal bone. In 1998, Esposito and colleagues critiqued the methods used to evaluate implant success, highlighting the challenges in accurately measuring losses below 0.2 mm and noting that assessment is primarily feasible for the mesial and distal aspects of implants. Nevertheless, this criticism loses validity when considering the evaluation of a consecutive series of at least 50 implants, as errors in radiographic measurements could either overstate or understate actual readings [13].

To sum up, there are several studies available that examine treatment outcomes after a decade, providing crucial insightinto the biological effects of implants. Wennerberg and colleagues recently synthesized these long-term findings, identifying 35 prospective and 27 retrospective studies spanning 10 years [14]. These studies encompassed five distinct implant systems, all demonstrating failure rates below 5%. Currently, there is no universalstandard for defining implant success or establishing specific parameter values indicative of implanthealth status, prognosis, or the necessity for clinical intervention. Nevertheless, it appears that the criteria established by Albrektsson and others, focusing on implant stability and radiographically determined crestal bone levels, remain the predominant factors when evaluating implant effectiveness [11].

### 4.2. Success Criteria of Zygomatic Implants—ORIS

Practitioners who deal with zygomatic implants agree with the statement that the criteria forsuccess for traditional implants are not the same for zygomatic implants. Therefore, Aparicio et al. suggested four different aspects: offset of the definitive prostheses, rhinosinusitis condition, soft tissue infection, and stability [7]. Assessing the criteria allows a practitioner to classify a patient into one of five conditionsas follows:

- Condition I: the optimal stage;
- Condition II: an alteration of routine without clinical impact;
- Condition III: a borderline situation with alterations that are clinically manifested but are still possible to successfully treat;
- Condition IV: the surviving implant supports the prosthesis but has not been measured according to the proposed criteria;
- Condition V: implant failure.

#### 4.2.1. Offset of the Prostheses (O)

**Figure 9:**
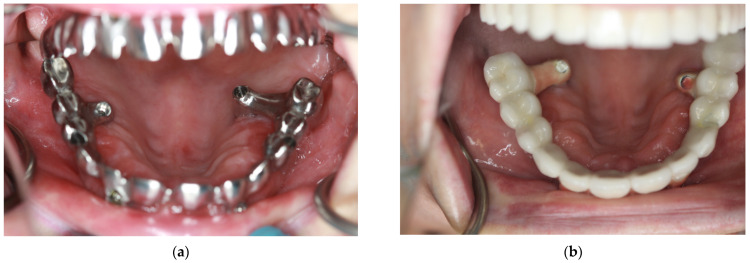
Implants screwed too palatally in region 16 and 26. In (**a**) milled metal to check bite and (**b**) final prosthodontic restoration.

**Figure 10:**
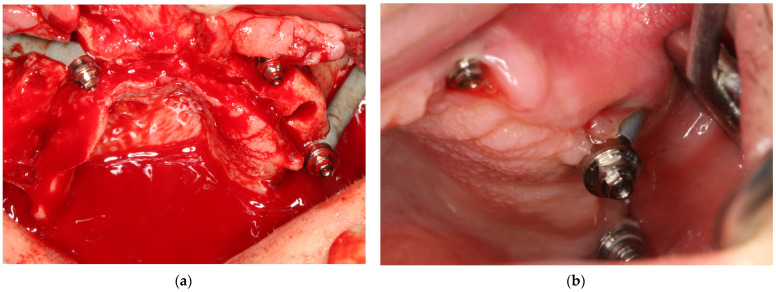
Implant in Region 26 screwed too buccally (**a**) resulted in implant removal 2 years aftersurgery (**b**)—lack of keratinized mucosa caused implant dehiscence.

The emergence of zygomatic implants in the palate can result in the creation of prostheses with “piping”. Occasionally, the presence of a sizeable dental bridge on the palatal side can cause discomfort and affect speech and hygiene accessibility(Figure 9). On the other hand, screwing the implant too buccally might be the reason for zygomatic implant loss(Figure 10).

#### 4.2.2. Rhinosinus Status (R)

It is important to evaluate the condition of the sinuses through clinical and radiographic examinations, as outlined in the Lanza and Kennedy survey (L–K survey) (Table 4), as well as the Lund–Mackay system [15,16].

#### 4.2.3. Peri-Implant Soft Tissue Condition (I)

**Figure 11:**
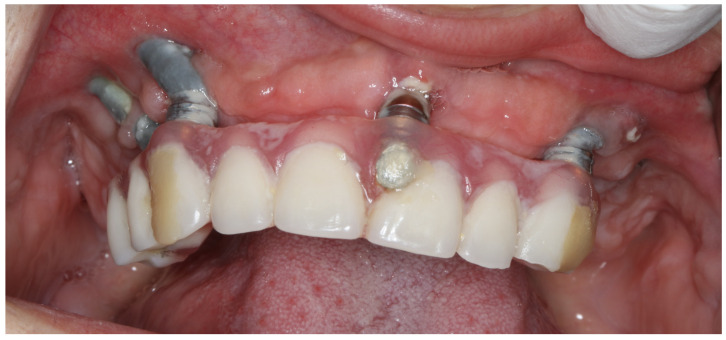
16 months after surgery with immediate implantation and immediate loading. The 31-year-old patient has not come to a check-up visit since surgery. Lack of keratinized mucosa around the implant neck, bacteria plaque, and rough implant surface resulted in plural dehiscences; however, the stability of implants was high. Loss of zygomatic implants is just a matter of a few years.

Exposure of the implant due to soft tissue dehiscence results in the partial visibility of the implant and the uncovering of the delicate bone layer located between the implant neck and the sinus cavity. The exposed bone undergoes more active remodeling over time, typically without causing pain. Should this condition persist in the critical area where bone thickness is minimal, it could lead to the formation of a connection with the sinus. The accumulation of bacterial film, particularly heightened in implants with threads or rough surfaces, could contribute to ongoing inflammation of the soft tissue. This inflammation could exacerbate the situation by accelerating bone remodeling and giving rise to subsequent complications, including sinus communications, aesthetic concerns, mucositis, or even cellulitis, as illustrated in Figure 11. 

#### 4.2.4. Stability (S)

The widely accepted method for confirming osseointegration is the clinical mobility test, which has also been introduced for zygomatic implants. However, varying levels of implant stability can be observed while examining a zygomatic implant. In some cases, exerting non-axial forces on an externally positioned zygomatic implant that is anchored in a low-quality zygomatic bone might result in minimal movement. Importantly, this movement does not necessarily correlate with clinical symptoms or pathological indications. This movement occurs due to the bone’s elasticity opposing the lateral force exerted externally on the extended implant head, which undergoes bending stress. Such movement should not be deemed problematic if there are no additional symptoms, and it should subside after attaching the superstructure with screws. To summarize, slight painless mobility could arise due to the elastic characteristics of the anchoring zygomatic bone when the implant experiences lateral loading from an external force. A Grade I success signifies no observable movement, Grade II implies minimal observable movement, and Grade III represents noticeable movement without signs of disosseointegration. Conversely, failure involves visible movement combined with rotational motion or discomfort. Any rotational movement of implants should be interpreted as an indicator of implant loss regardless of the accompanying pain.

ORIS scale seems to be the most relevant to assess zygomatic implant success. 

### 4.3. Intra-/Extrasinusand Intra/Extramaxillae

**Figure 12:**
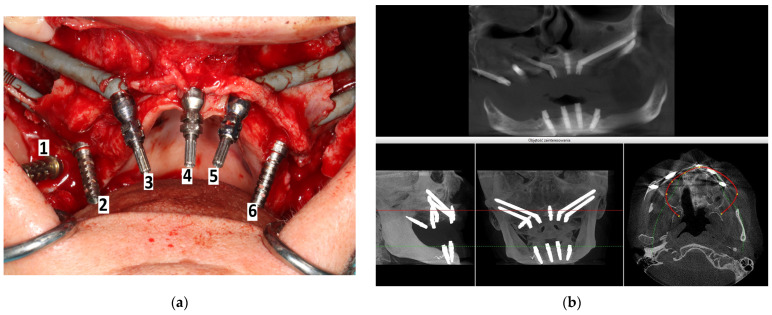
(**a**) Intrasurgical implant placement legend: 1—pterygoid; 2—intramaxillary and intrasinus; 3—extramaxillary and extrasinus; 4—traditional endosseous; 5—intramaxillary and extrasinus; 6—intramaxillary and intrasinus. (**b**) The same patient on CBCT taken on the day of surgery.

**Figure 13:**
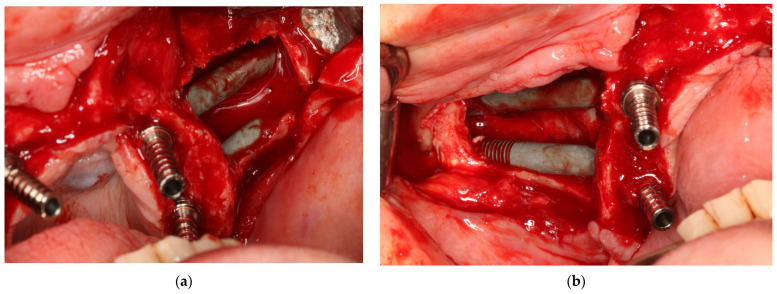
2 zygomatic implants on the left side placed (**a**)intramaxillary and (**b**) intrasinus, and the same implant placement in the same patient on the right side.

**Figure 14:**
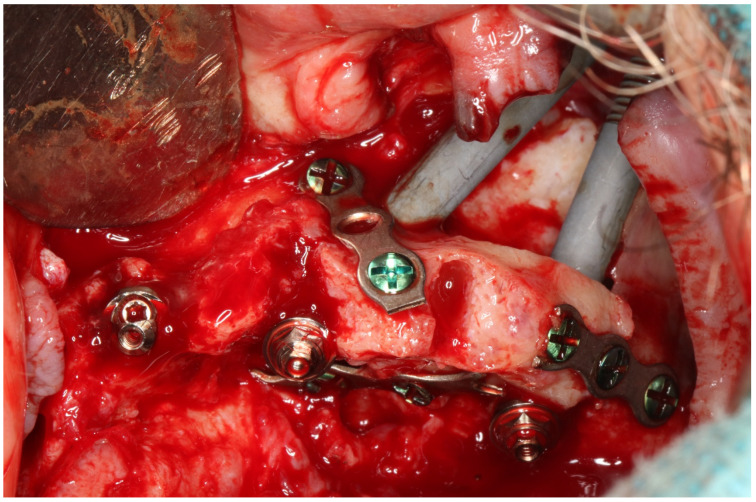
During the screwing of the zygomatic implant block, the alveolar process of the maxilla was chipped out because the zygomatic implant transfer was wider than the zygomatic implant neck. Thanks to the osteosynthesis plate by means of 2.0 self-tapping screws, the alveolar process was preserved, and implants were registered as being placed intramaxillary. It has been healed successfully.

There are numerous methods for screwing zygomatic implants, including the individualized method according to the particular anatomy of the maxilla zygomatic buttress and maxilla, i.e., the ZAGA concept [17,18,19,20,21,22,23]. Figure 12, Figure 13 and Figure 14 show how zygomatic implants look intraoperatively in the abovementioned places.

In the traditional Branemark method, zygomatic implants were screwed intrasinusally and intramaxillary. A bone was removed from the anterior and lateral wall of the maxilla sinus, approximately 10 mm in diameter. Thanks to the window, the desired path is created for the zygomatic implant from the sinus floor to the zygomatic recess of the maxillary sinus. The Scheiderianmembrane is gently dissected, freed from the sinus walls, and placed deeper. A few drillings areused to penetrate the alveolar process and the zygomatic bone. The estimated length of the zygomatic implant is selected using a depth gauge.

**Figure 15:**
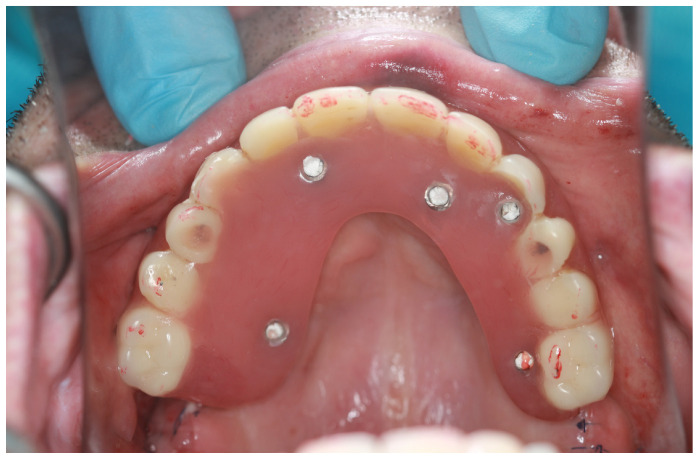
In severe maxilla defects, the placement of zygomatic intrasinus implants is a mistake for prosthodontic reasons. In the patient above, despite the extramaxillary approach, difficulties with speech and maintaining hygiene might occur.

In the article, all zygomatic implants were divided into intra- and extrasinus and intra- and extramaxillae placement. The preferable method of zygomatic implant placement was Stella’s method of trying to preserve the alveolar process bone around the zygomatic implant neck, so, given that prosthodontic screws were supposed to be on the occlusal surface of teeth, an intramaxillary approach was the method of choice. However, when a patient had a severe maxilla defect, then preparing osteotomy in the maxilla and the zygomatic bone for an implant demanded an extramaxillary approach. It was made to omit the palatal emergence of the implant head (Figure 14). The outcome of the palatal set of the head of the zygomatic implant might be speech hardships and discomfort with maintaining oral hygiene (Figure 15) [20,24,25,26]. Retrospectively, ZAGA division was also introduced to compare zygomatic implant placement with different parameters. 

### 4.4. Zygomatic Orbital Floor (ZOF)

**Figure 16:**
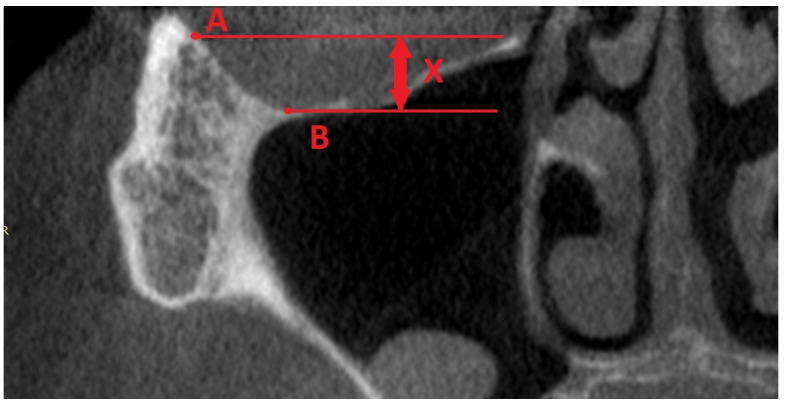
Zygomatic Orbital Floor (ZOF) classification. The most lateral point (A) was in the lower wall of the orbit. Reference points in the frontal plane on CBCT were the lowest (B). Then, two simple parallel lines perpendicular to the basal line of the transversal plane were prolonged, and the distance between them (x). Thanks to ZOF classification the risk of orbital damage is significantly lower.

One of the most dangerous complications in zygomatic implants is orbit penetration by a drill or by an implant. The anatomy of the zygomatic and orbit area must always be considered individually. ZOF is the best parameter, thanks to which a practitioner could assess the undercut of the lower and lateral wall of the orbit. The most lateral point (A) was in the lower wall of the orbit. Reference points in the frontal plane on CBCT were the lowest (B). Then, two simple parallel lines perpendicular to the basal line of the transversal plane were prolonged, and the distance between them (x) was the author’s intervention—ZOF (Figure 16).

ZOF classification was divided into four classes:

Class I—0–3 mm—flat;

Class II—4–6 mm—moderate;

Class III—7–9 mm—deep;

Class IV—more than 10 mm—extremely deep.

### 4.5. Limitations

Some patients who reported for consultation were burdened with a disease such as diabetes mellitus (>125 mmol/L). This group of patients was excluded from surgery. Moreover, smokers who were not able to give up at least 6 weeks before the surgery were not considered. Financialreasons also excluded patients from surgery. If these procedures were performed in a hospital and it was possible to hospitalize a patient, then patients with diabetes mellitus could probably be operated on, but in ambulatory conditions such as the dental clinic, it would not be safe.

## 5. Conclusions

The most important point in the procedure of the placement of zygomatic implants is the clinical mastery of the techniques for this surgical approach, which determinesthe success of the treatment. Thus, the described scales—ZAGA, ZOF, and ORIS—are unbiased criteria that could help practitioners treat patients better.

The ZAGA and ZOF scales are practical and valuable factors that should be taken into consideration before surgery, whereas to date, no better criteria have been described than the ORIS scalein terms of the success of zygomatic implants. The ZOF scale might omit perforation of orbit because the parameter warns a practitioner to be aware of the anatomy of the orbit, especially in the orbit’s lower wall. 

Many factors, including gender, intra or extrasinus, or intra or extramaxillary implant placement, are not statistically significant in terms of ZAGA, ZOF, or ORIS scale. However, some are significant, such as different types of surgeries with zygomatic implants, age, and height of zygomatic bone.

## Figures and Tables

**Table 1:** 81 patients were divided into four separate groups.

	Group 1	Group 2	Group 3	Group 4
Type of surgery:	4 zygomatic implants + 1 or 2 conventional implants	4 zygomatic implants	2 zygomatic + 4 conventional implants	3 zygomatic + 1 or 2 conventional implants
Number of patients:	14	15	46	6

Data of ZAGA class (Table 2) and ORIS (Table 3) were registered.

**Table 2:** ZAGA class was registered for all 81 patients on each side.

Maxilla Side	Number of Patients	Average of ZAGA Class	±	SD	Median	Minimum	Maximum
Right	81	2.00	±	0.91	2.00	0.00	4.00
Left	81	1.77	±	0.87	2.00	0.00	4.00

**Table 3:** ORIS class was registered for all 81 patients for each region of the zygomatic implant.

Regio of Zygomatic Implant	Number of Patients	Average of ORIS	±	SD	Median	Minimum	Maximum
16	81	1.28	±	0.79	1.00	1.00	5.00
26	81	1.31	±	0.74	1.00	1.00	5.00
13	33	1.33	±	0.69	1.00	1.00	3.00
23	33	1.45	±	0.94	1.00	1.00	5.00

**Table 4:** Lanza and Kennedy table showing rhinosinusitis criteria necessary for assessment for ORIS.

Lanza and Kennedy Task Force on Rhinosinusitis Criteria for the Diagnosis of Rhinosinusitis		
Major Criteria	Minor Criteria	Diagnosis of Rhinosinusitis Requirements
Facial pain or pressure	Headache	Two or more major criteria
Facial congestion or fullness	Fever (non-acute)	One major and two or more
Nasal obstruction	Halitosis	Purulence on nasal examination
Purulent discharge	Fatigue	
Hyposmia or anosmia	Dental pain	
Purulence on examination	Cough	
Fever (acute only)	Otalgia or aural fullness	
